# Prisons as Reservoir for Community Transmission of Tuberculosis, Brazil

**DOI:** 10.3201/eid2103.140896

**Published:** 2015-03

**Authors:** Flávia P.C. Sacchi, Renata M. Praça, Mariana B. Tatara, Vera Simonsen, Lucilaine Ferrazoli, Mariana G. Croda, Philip N. Suffys, Albert I. Ko, Jason R. Andrews, Julio Croda

**Affiliations:** Federal University of Grande Dourados, Dourados, Brazil (F.P.C. Sacchi, R.M. Praça, M.B. Tatara, M.G. Croda, J. Croda);; Adolfo Lutz Institute, São Paulo, Brazil (V. Simonsen, L. Ferrazoli);; Oswaldo Cruz Foundation, Rio de Janeiro, Brazil (P.N. Suffys);; Yale University School of Public Health, New Haven, Connecticut, USA (A.I. Ko);; Stanford University School of Medicine, Stanford, California, USA (J.R. Andrews)

**Keywords:** tuberculosis and other mycobacteria, *Mycobacterium tuberculosis*, bacteria, prisoners, reservoir, community transmission, genotyping, case–control study, epidemiology, Brazil

## Abstract

We conducted a population-based study of tuberculosis (TB) cases in Dourados, Brazil, to assess the relationship between incarceration and TB in the general population. Incarceration was associated with TB in an urban population; 54% of *Mycobacterium tuberculosis* strains were related to strains from persons in prisons. TB control in prisons is critical for reducing disease prevalence.

Brazil has the fourth largest incarcerated population in the world and a tuberculosis (TB) incidence that is 20 times higher among prisoners than among the general population (*1**,**2*). It has been hypothesized that prisons serve as institutional amplifiers for TB, wherein poorly controlled transmission among incarcerated persons is a driver of TB in the broader population (*3**,**4*). However, few data show for linkages between prison and community epidemics of TB. To address this issue, we conducted a population-based study of TB cases in Dourados, a medium-size city in west–central Brazil, and used case–control and molecular methods to assess the relationship between incarceration and TB in the general population.

## The Study

Dourados has a population of ≈177,160 persons, of which 1,500 are inmates of a prison for men. We identified and recruited TB patients reported to the Sistema de Informação de Agravos de Notificação National (Notifiable Diseases Information System) and who resided in Dourados during June 2009–March 2013. We then conducted a case–control study in which 2 control persons without a TB diagnosis were identified and matched for each TB case-patient according to age group and place of residence.

We performed conditional logistic regression to identify significant (p<0.05) risk factors for active TB. Variables were included in a multivariable model if they reached a significance level of p<0.20 in univariate analysis. *Mycobacterium tuberculosis* isolates were typed by IS*6110* restriction fragment length polymorphism (RFLP) analysis (*5*). RFLP patterns were analyzed by using an IS*6110* RFLP database (RIVM–Bionumerics; Applied Maths, Sint-Martens-Latem, Belgium). A cluster was defined as a group of ≥2 isolates obtained from different patients for which the RFLP patterns were identical with respect to the number and size of bands.

**Figure 1:**
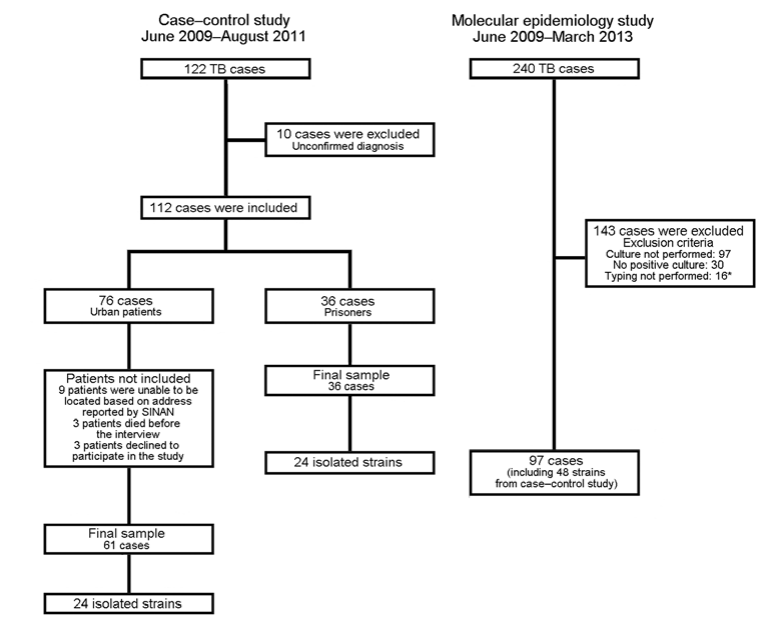
Flowchart for recruitment of patients with tuberculosis (TB) for case–control and molecular studies of prisons as reservoir for community transmission of tuberculosis, Brazil, June 2009–March 2013. *Eleven strains were not reactive after freezing, and 5 strains had <5 bands of IS*6110*. SINAN, Sistema de Informação de Agravos de Notificação (National Notifiable Diseases Information System).

A total of 240 TB cases were reported, of which 60 (25%) and 180 (75%) were in prisoners and community residents, respectively (Figure 1). The annual incidence of TB in the prisoner population was ≈40 times higher than in the community population (1,044 cases/100,000 persons [95% CI 797–1,344 cases/100,000 persons] vs. 26 cases/100,000 persons [95% CI 23–31 cases/100,000 persons]). All 60 prisoners had pulmonary TB and it was confirmed bacteriologically for 54 (90%) persons. Among 180 persons with TB cases in the community population, 133 (74%) had pulmonary TB, 34 (19%) had extrapulmonary TB, and 13 (7%) had both forms; 107 (59%) of the TB cases were confirmed bacteriologically. During the study, 49 (82%) prisoners with TB completed treatment, 2 (3%) were not cured, 3 (5%) died, and 6 (10%) were transferred to other prisons. Prisoners with cases were incarcerated for an average duration of 26 months before diagnosis.

We recruited 61 persons with TB and 122 controls from the community to evaluate risk factors for TB acquisition (Figure 1). Multivariable analysis showed that male sex (adjusted odds ratio [AOR] 6.6, 95% CI 2.4–18.1), monthly income ≤100 US dollars (AOR 3.4, 95% CI 1.1–10.6), alcohol use (AOR 11.5, 95% CI 2.0–67.0), known history of contact with a TB patient (AOR 5.6, 95% CI 1.4–22.0), and prior incarceration (AOR 24.5, 95% CI 2.4–254.6) were independent risk factors for TB (Table). A total of 23% (14/61) of the community cases were in persons previously incarcerated in the Dourados Prison.

**Table:** Risk factors for TB in community and prison populations, Dourados, Brazil, June 2009–August 2011*

Variable	Community, n = 183, no. asked/no.responded (%)					Prison, n = 108, no. asked/no. responded (%)		
	TB cases, n = 61	Controls, n = 122	Crude OR (95% CI)	Adjusted OR (95% CI)		TB cases, n = 36	Controls, n = 72	Crude OR (95% CI)
Male sex	41/61 (67)	42/122 (34)	3.9 (2.0–7.5)	6.6 (2.4–18.1)		NA	NA	NA
Income ≤$100†	19/61 (31)	22/122 (18)	2.1 (1.0–4.2)	3.4 (1.1–10.6)		NA	NA	NA
No primary school	15/61 (25)	25/122 (21)	1.3 (0.6–2.6)	NA		11/36 (31)	14/72 (19)	1.82 (0.73–4.57)
Smoked	20/61 (33)	24/122 (20)	2.0 (1.0–4.0)	NA		17/36 (47)	36/72 (50)	0.89 (0.40–1.99)
Alcohol use	14/61 (23)	7/122 (6)	4.9 (1.9–12.9)	11.5 (2.0–67.0)		6/36 (17)	12/72 (17)	1.00 (0.34–2.93)
Drug use	15/61 (25)	2/122 (2)	19.6 (4.3–88.9)	NA		26/36 (72)	48/72 (67)	1.30 (0.54–3.13)
Diabetes	7/61 (12)	13/122 (11)	1.1 (0.4–2.9)	NA		1/36 (3)	1/72 (1)	2.03 (0.12–33.40)
Contact with person with TB	18/61 (30)	15/122 (12)	3.0 (1.4–6.5)	5.6 (1.4–22.0)		23/36 (64)	54/72 (75)	0.59 (0.25–1.40)
*Mycobacterium bovis* BCG vaccine scar	41/61 (67)	96/122 (79)	0.6 (0.3–1.1)	NA		28/36 (78)	54/72 (75)	1.17 (0.45–3.02)
Prior incarceration	14/61 (23)	1/122 (0.8)	36.0 (4.6–281.8)	24.5 (2.4–254.6)		NA	NA	NA

*TB, tuberculosis; OR, odds ratio; NA, not applicable; BCG, Bacillus Calmette-Guérin. †Monthly individual income in US dollars.

**Figure 2:**
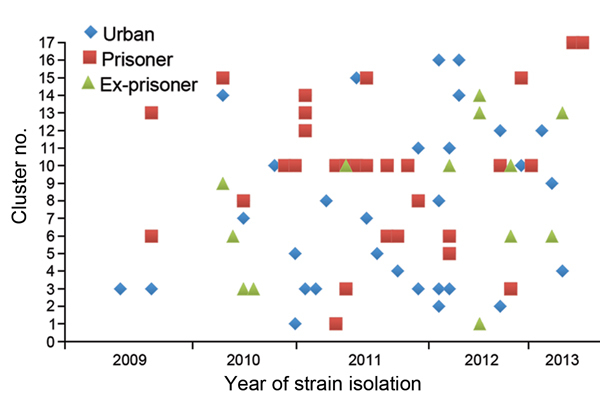
Temporal distribution of *Mycobacterium tuberculosis* strains isolated from the urban population, prisoners, and ex-prisoners in Dourados, Brazil, clustered by IS*6110* restriction fragment length polymorphism analysis, June 2009–March 2013, and stratified by year of isolation and number of the identified cluster.

We genotyped 97 (86%) of 113 strains isolated from persons with culture-positive *M.* *tuberculosis* infection, of which 59 and 38 were isolated from community persons and prison patients, respectively (Figure 1). Of these, 79 (81%) isolates were grouped into 17 clusters, and 18 isolates had unique RFLP patterns. Among the 17 cluster types, 10 types included 65 strains from community and prison settings, 6 types included 12 strains exclusively from the community setting, and 1 type included 2 strains exclusively from the prison setting. Cluster 10, the largest cluster (20 cases), was predominantly found in prisoners, but was also isolated from ex-prisoners and community members without a history of incarceration. Of the 12 community cases with cluster types exclusively found in the community, one was in an ex-prisoner (Figure 2).

Overall, 54% (32/59) of community strains belonged to cluster types that also included prison strains. Among the 32 cluster strains that were circulating in the prison and community, 12 (37%) were isolated from ex-prisoners who were recently released from prison. Ten (83%) of 12 cases occurred within ≤2 years of the inmate’s release from prison (Figure 2).

## Conclusions

Prisons have long been recognized as high-risk environments for TB (*6**,**7*), but there are little data concerning the potential transmission of the disease into community settings. During a 4-year period in a medium-size city in Brazil, 25% of TB cases occurred among prisoners, who represented <1% of the population. Our case–control study showed that that ex-prisoners had 23% more cases of TB than the general population. Among cases in ex-prisoners, 83% (10/12) were diagnosed in the first 2 years after release from prison, which suggests recent infection acquired in the prison setting.

Although exposure to TB might occur after a prisoners’s release, we believe that this is less likely because most (71%) ex-prisoners had isolates with the same RFLP pattern as patterns found in prison isolates. Also, 83% (10/12) of ex-prisoners who had an isolate with a similar genetic profile were reported after a case of TB in the prison was reported (Figure 2). Furthermore, we found that baseline tuberculin skin test positivity rates were low (7%) among newly incarcerated inmates, which further supports the assertion that the high rate of TB among prisoners was caused by transmission in the prison setting, rather than by exposure to the disease in the community before incarceration.

The presence of multiple clusters involving prisoners and the general population indicates that TB can spread between these 2 populations. Only 1 previous study reported a link between TB cases in a community setting and those in a jail or prison, but this finding was based on an outbreak that involved only 1 strain (*8*). If one considers the genetic linkages observed in this study and the high rates of disease among prisoners and ex-prisoners, our findings suggest that prisons serve as major reservoirs of TB for the general population.

This study had several limitations. First, we did not evaluate the linkage between cases by investigating close contacts, which limited our ability to establish exact epidemiologic connections between patients. Future contact tracing studies might enhance our understanding of the chain of TB transmission in these settings (*9**–**11*). Second, we used RFLP to assign clusters, which might overestimate the proximity of genetic or epidemiologic linkages (*12*); future studies involving whole-genome sequencing might help clarify the timing and directionality of disease transmission (*13*). Third, we assessed data for only 1 city and 1 prison. The prison had typical conditions in terms of layout, crowding, and diagnostic resources. However, other studies have found that TB incidence and transmission rates are even higher in other prisons in Brazil (*3**,**14**,**15*). Thus, the contribution of spillover infections to the general community may be even greater.

Our data demonstrate that incarceration is a strong risk factor for acquiring TB and that the epidemic of TB in prisons is interlinked with that in the general population. Policies and programs aimed at reducing transmission in prisons and preventing TB among released prisoners should be considered to successfully control TB in the general population. Effective responses will require improving TB diagnostic capacity in prisons, implementing active case detection strategies, such as annual mass screening, testing for latent TB and provision of isoniazid preventive therapy, and developing transitional care and follow-up programs for prisoners released into the community.
